# Assessment of Water Quality Profile Using Numerical Modeling Approach in Major Climate Classes of Asia

**DOI:** 10.3390/ijerph15102258

**Published:** 2018-10-15

**Authors:** Muhammad Mazhar Iqbal, Muhammad Shoaib, Hafiz Umar Farid, Jung Lyul Lee

**Affiliations:** 1 Graduate School of Water Resources, Sungkyunkwan University, Suwon-si 2066, Korea; mazhar0559@skku.edu (M.M.I.); changezi@skku.edu (M.S.); 2 Department of Agricultural Engineering, Bahauddin Zakariya University, Multan 66000, Pakistan; hufarid@bzu.edu.pk

**Keywords:** Asia, water quality, climate classes, QUAL2Kw, WQI

## Abstract

A river water quality spatial profile has a diverse pattern of variation over different climatic regions. To comprehend this phenomenon, our study evaluated the spatial scale variation of the Water Quality Index (WQI). The study was carried out over four main climatic classes in Asia based on the Koppen-Geiger climate classification system: tropical, temperate, cold, and arid. The one-dimensional surface water quality model, QUAL2Kw was selected and compared for water quality simulations. Calibration and validation were separately performed for the model predictions over different climate classes. The accuracy of the water quality model was assessed using different statistical analyses. The spatial profile of WQI was calculated using model predictions based on dissolved oxygen (DO), biological oxygen demand (BOD), nitrate (NO_3_), and pH. The results showed that there is a smaller longitudinal variation of WQI in the cold climatic regions than other regions, which does not change the status of WQI. Streams from arid, temperate, and tropical climatic regions show a decreasing trend of DO with respect to the longitudinal profiles of main river flows. Since this study found that each climate zone has the different impact on DO dynamics such as reaeration rate, reoxygenation, and oxygen solubility. The outcomes obtained in this study are expected to provide the impetus for developing a strategy for the viable improvement of the water environment.

## 1. Introduction

Climate characteristics have a significant impact on the spatio-temporal variation of surface water quality. Deteriorating water quality presents a serious problem to the water security of many zones in different climatic classes. The geo-spatial variability of water resources and socio-economic pressures present complex challenges to sustainable management of water resource to meet domestic, agricultural, and industrial water demands [1]. The impact of climate change on water stressors has compounded further the challenge of the planning and prediction of water quantity and quality within the hydro-ecosystems in different climate regions. Some studies focusing on the impact of climate change on hydrological systems, while much attention has been given to water balance, less attention has been given to trans-climatic water quality profiles [2,3]. It has been acknowledged that the impact of climate change will influence the quality of water through human activities and complex natural mechanisms over different regions [4,5,6]. Variation in climate may also have an indirect influence on surface water health through changes in land scape pattern [7,8,9,10]. Accordingly, a deterioration of water quality has been observed in a number of studies on the influence of water quality due to climate change, particularly on water pollutants and their physio-chemical characteristics—such as DO, total suspended solid (TSS), ammonia (NH_3_), and turbidity—within the changing hydrometeorological ecosystems [11,12,13]. Surface water quality has a diverse pattern of variations and it is not only a function of climate but also urbanization through anthropogenic activities [13,14,15].

Surface water quality has considerable influence on an organism’s wellbeing and on global economic and social advancement. Conversely, regional climate, human actions, and economic and social activities have various effects on water quality [16,17,18]. In water quality simulations, it has been observed that the water quality models and various artificial intelligence approaches provided reasonable results and the practical modeling approach guarantees the use of models for forthcoming options for surface water quality [19,20,21,22,23]. In the last few decades, the development of surface water quality models (WQMs) to support decision making processes in water management and governance has intensified. From simple empirical based models to advanced and complexly coupled models, numerous WQMs have been developed for the analysis and prediction of the quality of water due to changes in rivers, lakes, and oceans. The WQMs provide extensive waterscape analytic and complex geo-morphological simulation capabilities [24]. Most of the models are adaptable to various environments subject to appropriate definitions of boundary conditions, dimensional variation, and parameter characterization. Basic models require hydro-meteorological data inputs, including precipitation, flow, humidity, and chemical and biological data with varying temporal resolutions [19,25,26]. Good WQMs are expected to process higher temporal resolution results from the lower resolution of the corresponding input data available [27]. The increasing demand for modeling integrated physio-chemical and hydro-biological processes in typical ecosystems requires the inclusion of additional fluxes to simulate mutually dependent complex processes such as nutrient generation, transport, transformation, and recirculation in hydrological systems. Such high expectation output from the advanced and coupled WQM models is limited due to over-parameterization and associated assumptions in the process [28,29]. Opinions remain divided on whether higher dimensional complex models (two-dimensional or three-dimensional) or simple models based on appropriate theories and logic is the best approach to water quality modeling [30,31,32].

In this context, QUAL2kw is the prominent model for the simulation and comparison of water quality and quantity in diverse settings for various parameters [33,34]. The QUAL2kw is a flexible one-dimensional (1-D) dynamic model having two version that can be applied also to steady state and unsteady state mode. It has several new elements, including DO dynamics with rooted plants, changing of algal extinction to BOD (CBOD), and utilization of CBOD in the denitrification process [35]. QUAL2Kw has been applied in many settings [33,34,36,37,38,39]. QUAL2kw can model the compound interaction between and across organic and inorganic pathways in aquatic conditions. It can capture reactions and the decay process of phytoplankton, nitrate, ammonia, phosphate, DO, BOD, organic phosphorous, and nitrogen concentrations [34,40,41,42]. The dynamic interactions between nutrient loads from various sources and the consequent longitudinal and lateral water quality pattern in the recipient water body are best described by the WQMs [27,31].

The water quality index (WQI) is used to evaluate the quality of the water ecosystem from different sources using a group of selected parameters. It reduces the long list of parameters to a single composite number, normally dimensionless, in a simplistic reproducible sequence [43,44,45,46]. The WQI has been widely applied in the monitoring of water quality for both groundwater and surface water, specifically on rivers, playing a significant role in water resource management [47,48,49]. WQI can be developed over the longitudinal profile of a river using the corresponding water quality results obtained from the best WQMs to indicate the spatial trends of water quality along a river regime [41,50]. Compared with the conventional water quality surveys, WQI methods are an efficient tool of communication and facilitate understanding of the overall state of the water body based on a single value (index) rather than the list of parametric values. Thus, rather than comparing the various values for different parameters, WQI is an efficient and effective way to describe and easily compare the characteristic state of water quality, which is crucial in water resource management.

Several reviews and studies have assessed the impacts of climate on hydrological circulation in several regions [51]. Currently, many studies and literature reviews have been carried out on climate change phenomena and sustainable urban water supply, at local and global scales, outlining the problems associated with climate change impacts and uncontrolled urbanization [5,10,48,49]. The mitigation of the effects of climate change requires a collection of reliable data on water quality for effective monitoring and management at local, regional, and global levels as previously performed in several countries [48,49,52,53,54]. However, a gap remains on the longitudinal profiling of water quality assessment across the different climatic regions. The usual method of water quality conservation involves the use of sustainable ecosystem health strategies, allowing for the assortment of pollutant discharges and flow control but not allowing for the assessment of environmental factors [55].

Previous studies have revealed that DO concentrations in rivers are influenced by environmental conditions upstream points and along the sections of the river [20]. At the same time, different studies discuss how higher temperatures reduce the solubility of DO and re-oxygenation rate. Consequently, it is critical for environmental researchers to consider the regional climate impact independently on river water quality along with other management strategies such as flow augmentation, aeration, and water treatment practices [20,56,57,58]. This assessment is used to evaluate the spatial variation of water quality over different Koppen climate classes. The following study was carried out within the main climatic zones of Asia. Streams having similar characteristics were selected from each climatic class. The main limitation experienced in this study is; scarcity of long-term data, especially for the Baghmati river and Galing river. In each climate zone, there are several rivers, therefore difficult to obtain sufficient long-term data for each river that may be identified for the study.

The present work explores the complex interplay of WQI among four different climatic classes in Asia and the performance of the model is evaluated. Through analysis, it is determined whether the spatial scale variations are consistent or differ within each climatic class. In this study, the Koppen-Geiger climate classification was used to select the four major climatic regions over Asia. Koppen-Geiger climate classification comprised a total of five main climatic regions according to climate boundary condition [59,60]. Out of the five main climatic regions, four major regions were selected covering the entire study area, Asia. To this aim, the objectives of this research are: (1) to predict the longitudinal water quality profile over different climate classes; (2) to develop WQI for different streams having different climatic patterns; (3) to investigate the spatial scale variation of the river WQI over different climate classes; and (4) to investigate the spatial scale relationship of the river water quality and flow toward the downstream end of the river profile over different climate classes.

## 2. Materials and Methods

### 2.1. Study Site

This research was conducted in four main climatic regions of Asia, arid, temperate, tropical, and cold, based on the Koppen-Geiger climate classification. Many authors have used the original Köppen climate classification to determine the climate zones and to analyze their findings in specific climatic zones [61,62,63]. The Koppen climate classification has been modified by various authors [64,65,66], but the original Koppen climate classification (hereafter referred to as Koppen-Geiger climate classification), is still the most frequently used classification [52]. Four different rivers having similar characteristics were selected from each climatic class: Yamuna River, India from the arid climatic region; Baghmati River, Nepal from the temperate climatic region; Galing river, Malaysia from the tropical climatic region; and Nakdon River, Korea from the cold climatic region. The Köppen climate classification system is the most extensively used system for classifying the world’s climates. However, it has been updating regularly since it was first published in 1884. In the current study, the authors have used the most recent updated climate classification map [64]. There are many rivers, lakes, streams, creeks, and artificial channels in Asia. However, in the present study, the authors used these rivers based on the facts that.

- The study selected the rivers with the comparably similar hydraulic characteristics, having a natural flow, unlike artificial channels.
- All the rivers have mostly similar land type characteristics passing through urban as well as vegetative areas.
- All the rivers section having several urban networks of wastewater drains flowing into them.
- All the rivers have common data characteristics which are useful for their comparative water quality profile analysis and assessment.

**Figure 1:**
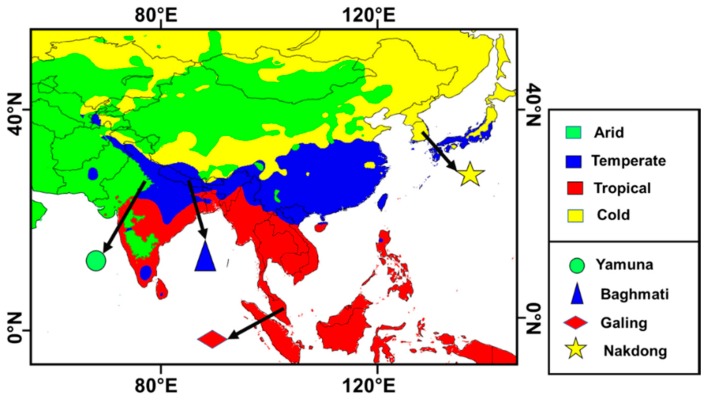
Locations of four different rivers and their corresponding major Koppen-Geiger climate classes.

Furthermore, we have to choose one stream from each climate classes and the rivers which have been chosen for this study have common temporal data. Moreover, the chosen rivers individually, have been part of the recent studies, therefore it was useful to compare our results with those published studies. Therefore, based on the above-mentioned facts, the authors chose these rivers for their study. Figure 1 describes the study area and four majors corresponding Koppen-Geiger climate classification regions.

#### 2.1.1. Yamuna River

Yamuna River in India is located in the arid climatic region. It is one of the main tributaries of the river Ganges and is used as the main source of drinking water for over 16.8 million people in New Delhi [67]. The condition of the river water quality deteriorates once the river flows out of the city. Even though the national capital region constitutes only 1% of the watershed, it generates more than half of the pollution load into the river [68]. The stretch of the river within the city is not only necessary for both ecological and economic development but acts as a sink for waste-disposal and transport. Moreover, unplanned development and population pressure within the national capital territory have exerted stress on water supply and sanitation of Yamuna River. Lack of sufficient sewer connectivity among waste water treatment plants and surface drains caused a direct discharge of waste water into the Yamuna River, leading to increased effluent load into the river [69].

**Figure 2:**
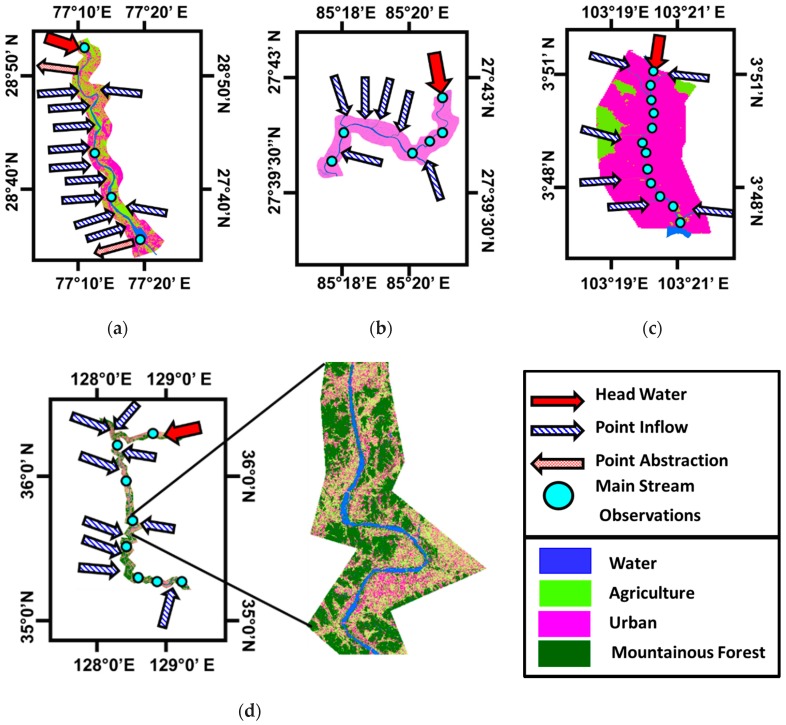
Description of study area (**a**) Yamuna River, India; (**b**) Baghmati River, Nepal; (**c**) Galling River, Malaysia; and (**d**) Nakdong River, Korea.

The Yamuna River provides water for domestic and industrial use in Delhi, the capital city of India. The diversion canal for the water supply to the city of Delhi is located 10 km away, between Palla and Wazirabad. During the summer, the residual flow after the diversion remains for the environmental flow during summer seasons. After only 300 m downstream of the Wazirabad barrage, a large drain known as the Najafghar drain enters the waterway. Downstream of the Najafghr drain, 13 others small to medium drains also enter the Yamuna waterway. The waterway exits the city near the Okhla barrage, nearly 39 km downstream of Palla. The total area of the Yamuna basin is around 9500 Ha, of which almost 8000 Ha is a dry area which also contributes to the river pollution by direct surface runoff [70,71]. The major sources of the Yamuna water pollution are characterized as point sources through which contaminating refuse is discharged directly into the Yamuna River [72]. Figure 2a shows a detailed description of the Yamuna River in India.

#### 2.1.2. Baghmati River

The Baghmati river originates from the Sheopuri ranges at 1500 m above sea level (27°47′ N, 85°17′ E) in Nepal. It then flows through the Kathmandu valley in a highly populated area. Increased population pressure and the high rate of urbanization within the catchment have changed the water quality and the quantity dynamics of the Bagmati River [73] (Jain and Sinha 2004). In particular, during the dry periods, the river water quality deteriorates, limiting any meaningful social and economic use [74,75]. The mean annual precipitation in the predominantly alluvial soil prairies of the river basin is 1250 mm, of which nearly 1120 mm falls during the summer monsoon period. The source of the river (foothills regions) receives much higher precipitation (greater than 2000 mm yearly) [76]. The study site is a 25 km stretch of the river meandering through the Kathmandu valley in Nepal. The stretch rises from about 25 km north of the capital city, Kathmandu, before flowing downwards to the valley floor, cutting through the Mahabharat ranges southwards before finally emerging onto the Ganges plains. For this study, the stretch from the Atterkhel location to Chovar village (the exit point from the valley) was identified. Kathmandu fulfilled around 90% of the water requirements during the wet season and almost 60% during the dry period. In addition, the river serves as a cultural resource, especially to the Hindu–Buddhist communities in Nepal [36,37]. Figure 2b shows the location of the headwater, point source inflow, and mainstream observation points of the Baghmati River, Nepal.

#### 2.1.3. Galing River

Similar to other global climatic zones, the temperate zone (i.e., Galing River in Malaysia) also has major water quality concerns. One of the main problems is the deteriorating water quality of Galing River, which flows through the locality of Kuantan, Pahang. Presently, the general quality of Galing River is very poor, and is described as Class IV based on the water quality standard of Malaysia [77]. The river water quality has primarily deteriorated due to pollutants discharged from both residential and commercial settlements without adequate treatment [78,79,80]. The Galing waterway is the prime drainage system for the entire metropolitan area of Kuantan, and it drifts through the furthermost built-up area of Kuantan city, sited in the eastern coastal region of the Malaysian peninsula. Kuantan is the capital city of the Pahang state and one of the largest cities in Malaysia. Over the past decade, due to the enactment of the “Kuantan District Locality Plan 2004–2015”, the area has been developing very rapidly, resulting in major environmental and ecological poverty. The coastline area of Pahang state has been remarkably urbanized and forested, and agricultural sections have decreased in size and have been converted to land for residential and industrial buildings [77,78,79,81]. The river begins in the Semamb industrial zone and meets the Kuantan River at site 8 km upstream from the coast of the South Sea [77]. Figure 2c shows the location of the headwater, point source inflow, and mainstream observation points.

#### 2.1.4. Nakdong River

The area selected for the Nakdong River in this study is located between longitude 127°29′19″ E–129°18′00″ E and latitude 34°59′41″ N–37°12′52″ N in South Korea. The river meets the watersheds of the Seomjin river in the west, the Han river to the north and the coastal watershed in the east, facing the southward to the seafront of the Nakdong River in the south [82]. The Nakdong River has the second largest watershed, after Han river in the Korea South with an expansive watershed’s estimated to be 9196.18 square miles (which is about 25% area of the whole country) and the length of the river is about 522 km [83].

The Nakdong River is a network of one of the key water resources supporting major cities such as Daegu and Busan on the southeastern parts of South Korea [84]. The watershed of the Nakdong River comprises a woven network of 13 streams: Nakdong River, Geumho River, Gam Creek, Hwang River, West Nakdong River, Nam River, Pyeonggang Creek, Yangsan Creek, Miryang River, Naeseong Creek, Maekdo River, and Deokcheon River. Figure 2d shows the location of the headwater, the point source inflow, and the mainstream observation points of the Nakdong River, South Korea.

### 2.2. Input Data Sets

In this study, the average annual input data sets were utilized, including the initial concentrations of BOD, DO, pH, components of the nitrogenous compound, and point inflow information for the period of 2011 and 2012. Data for Nakdong River was obtained from the database systems of Korea, including the Water Environment Information System (WEIS) (http://water.nier.go.kr), Water Resources Management Information System (WRMIS) (http://www.wamis.go.kr) Korea Environment Corporation, and the Korea Meteorological Administration (KMA) (http://www.weather.go.kr). Data for the Yamuna River case were collected from various government agencies including the Central Water Commission (CWC) (http://www.cwc.nic.in/), Central Pollution Control Board (CPCB) (http://cpcb.nic.in), National Water Quality Monitoring Program (NWMP), and the Ministry of Environment, Forest, and Climate Change (MEFC) (http://www.moef.nic.in/). Input data for the case of Baghmati River were obtained from the Central Bureau of Statistics (CBS), the Department of Hydrology and Meteorology (DHM) (http://www.dhm.gov.np/), and other scientific papers, journals, and personal communication from various sources such as the Ministry of Urban Development (MUD) (http://www.bagmati.gov.np) as well as other scientific papers, journals, and personal communication from various sources. The input data for Galing River were gained from the Department of Environment (DOE) and the Ministry of Natural Resources & Environment (https://www.doe.gov.my), the Malaysia Environmental Performance Index (MEPI) (http://www.epi.utm.my) supplementary data information, and the Department of Irrigation and Drainage (http://h2o.water.gov.my), as well as other scientific papers, journals, and personal communication from various sources. Furthermore, the missing meteorological information including precipitation, wind speed, air temperature, solar radiation, and specific humidity was retrieved from the global data assimilation system (GLDAS) datasets [85]. The adopted parameters are mostly used as water quality indicators in many countries. Therefore, they were selected as performance indicators to examine the water quality profile. The nitrogenous compound such as TN, NO3, and NH3 have been used in our study as these compounds have the most severe impact on the aquatic environment. Furthermore, among all other nitrogenous compounds, these three most important components are available which are common for all streams. For example, organic nitrogen is available in case of the Nakdong river but it is not available in the case of other streams.

### 2.3. Water Quality Modeling

The one-dimensional stream water quality model, QUAL2Kw, was selected and compared for water quality simulations on a selected river of each climate class. Calibration and validation were performed for both models over the different climatic regions, separately.

#### QUAL2Kw

QUAL2Kw is a one-dimensional water quality analysis model commonly used in water resource assessments. It is suitable for application to an adequately-mixed river-condition with a comprehensive mass transport evaluation capacity to analyze the molecular diffusion, advection, and dispersion phenomena in one direction [35]. This model is used to simulate a river as a chain of numerical units having identical hydro-geometric properties and hydraulic characteristics, including biological and chemical rate constants. The QUAL2Kw algorithm is used in the Visual Basic for Applications (VBA) program language. MS Excel is used for the graphical user function for data entry, operations, and visualization of the output information. Fortran 95 program is used to execute numerical algorithms for the unit element processing sequence. The model is then run using a program compiled by the Excel VBA interphase. QUAL2Kw applies a mass conservation governing relation at a particular concentration *S_i_* in the elemental (without hypothetic) unit *i*. In this case, the transport process and input loading parametric-terms are excluded from the equation when considering the algae modeling [33,34,35,36,37,41].

(1) $$\frac{dS_{i}}{dt}=\frac{Q_{i-1}}{V_{i}}S_{i-1}-\frac{Q_{i}}{V_{i}}S_{i}-\frac{Q_{\mathrm{wd},i}}{V_{i}}S_{i}+\frac{E_{i-1}}{V_{i}}\left(S_{i-1}-S_{i}\right)+\frac{E_{i}}{V_{i}}\left(S_{i+1}-S_{i}\right)+\frac{W_{i}}{V_{i}}\pm K_{i}$$

**Figure 3:**
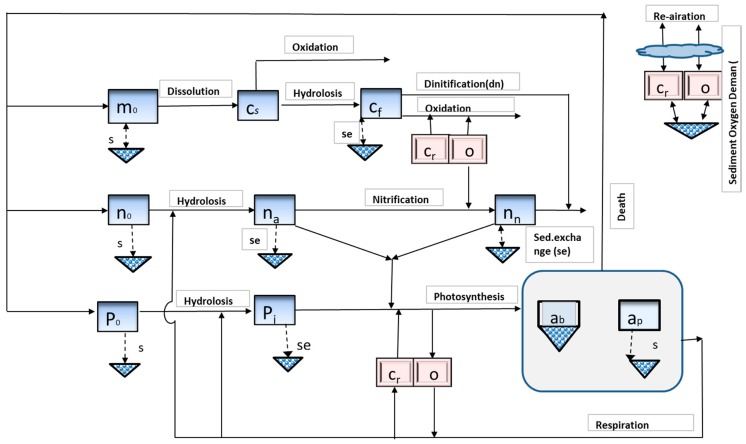
QUAL2Kw graphical illustration of water quality parameters: o—dissolved oxygen, a_p_—phytoplankton, a_b_—algae (bottom algae), n_o_—nitrogen (organic), M_o_—detritus, C_f_—CBOD fast, C_s_—CBOD slow, C_r_—inorganic carbon, n_n_—nitrite nitrogen, n_a_—ammonium nitrogen, p_i_—inorganic phosphorus, and p_o_—organic phosphorus.

Here, *S* is the concentration (mg/L), *Q* is the discharge (m^3^/s), *wd* is the withdrawal (m^3^/s), *V* is the flow quantity (m^3^), *E* is the coefficient of bulk dispersive flux among reach *i* and consecutive reach *i* + 1 (m^3^/s), and *W* is the external pollutant (mg/s), and *K* is the recipient sinks and discharge of external sources of the pollutant due to physio-chemical reactions and mass transport dynamics (g/m^3^/s). The sink/source terminology (*K*) in the equation needs a vast specification of the number of water quality parameters for every state variable (e.g., maximum production rate of phytoplankton). The user can select appropriate parameters as constants which are applied in an optimization of the genetic algorithm (GA). Further information about the model equations can be obtained from (https://www.epa.gov/). Figure 3 shows graphical illustration of the interactions water quality parameters involves in QUAL2kw model. The study adopted 1 D model because of the fact that the authors had only the longitudinal water quality data of all the four rivers. Furthermore, two-dimensional (2-D), or three-dimensional (3-D) water quality models are more suitable for water bodies having higher retention times such as lakes or ponds. Therefore, the authors were not unable to use a 2-D or 3-D water quality models. The other feasible alternative was QUAL2K. QUAL2Kw is the modified form of QUAL2K which has the ability to automatically optimize the data. Furthermore, QUAL2K and QUAL2Kw are both renown water quality models and over the years many researchers have used these models for their research.

### 2.4. Calibration and Validation of the Model

Calibration is essential for the consistency of the final model results to analyze water quality. The following steps were undertaken to calibrate the QUAL2Kw water quality models. Obtained data from each climatic region were simulated for a period of 2011 by repetitive tuning of reaction constants and environmental parameters (Tables S1–S4). The calibrated reaction constants and environmental parameters were used for verification of the model results. The boundary reference conditions—including BOD, DO, qmmonia nitrogen (NH_3_-N), nitrate nitrogen (NO_3_-N), and total phosphorus (T-P)—were used along the upstream as well as downstream sections and drains. The kinetic input parameters for the model were extracted from the literature and other models and were then adapted to the local conditions. The final output comprised a combination of the optimal states of every constant adjusted. The model calibration and the model validation were conducted in parallel.

### 2.5. Assessment of the Model Accuracy

The accuracy of model simulation is achieved by comparing the simulated data (theoretical) with the corresponding observations (actual). Ten statistical rating tools were used in this study to evaluate the accuracy of the model [86,87,88,89,90]. The first statistic tool used is the mean absolute error (MAE), which measures the deviation among predicted results and observed values. The MAE formula is given as

(2) $$\mathrm{MAE}=\frac{1}{N}\sum_{n=1}^{N}\left|D_{n}-M_{n}\right|$$

The second statistic tool used is the mean square error (MSE), which estimates the mean of the square of variation or errors among the model predictions and measured values.

(3) $$\mathrm{MSE}=\frac{1}{N}\sum_{n=1}^{N}{\left(D_{n}-M_{n}\right)}^{2}$$

The third statistic tool used is the root mean square (RMSE), which represents the standard deviations of samples among measured and predicted values. The units of the RMSE are the same as the units of the model predictions and ground observations.

(4) $$\mathrm{RMSE}=\sqrt{\frac{1}{N}\sum_{n=1}^{N}{\left(D_{n}-M_{n}\right)}^{2}}$$

The fourth statistic tool used is the normalized root mean square error (NRMSE), which is a measure of the deviation among observed and predicted results. As the error lessens, the model prediction accuracy rises.

(5) $$\mathrm{NRMSE}=\sqrt{\frac{1}{N}\sum_{n=1}^{N}{\left(D_{n}-M_{n}\right)}^{2}}\times \frac{1}{{\bar{D}}_{n}}$$

The fifth statistic tool used is the mean absolute percentage error (MAPE), measures the variation of the modeling results in percentage

(6) $$\mathrm{MAPE}=\frac{1}{N}\sum_{n=1}^{N}\left|\frac{D_{n}-M_{n}}{D_{n}}\right|$$

The sixth statistic tool used is the coefficient of determination (R^2^), which evaluates the relative deviation of simulated results from the observed data attained by the model. The expression for (R^2^) is obtained by squaring the Pearson correlation coefficient (PCC) equation as

(7) $$R^{2}=\frac{{\left\{\sum_{n=1}^{N}\left(D_{n}-\bar{D_{n}}\right)\left(M_{n}-\bar{M_{n}}\right)\right\}}^{2}}{\sum_{n=1}^{N}{\left(D_{n}-\bar{D_{n}}\right)}^{2}\sum_{n=1}^{N}{\left(M_{n}-\bar{M_{n}}\right)}^{2}}$$

The seventh statistic tool used is Nash–Sutcliffe’s model efficiency (ME), which measures the ratio of the model deviation from the true (measured) data [80].

(8) $$\mathrm{ME}=1-\frac{\sum_{n=1}^{N}{\left(D_{n}-M_{n}\right)}^{2}}{\sum_{n=1}^{N}{\left(D_{n}-\bar{D}\right)}^{2}}$$

The eighth statistic tool used is the percentage model (PM) bias, which is determined by the summation of the difference between the normalized model error normalized from the observed data in order to give a measure if the model under or overestimations against the observed values.

(9) $$\mathrm{PMbias}=\frac{\sum_{n=1}^{N}\left(D_{n}-M_{n}\right)}{\sum_{n=1}^{N}D_{n}}\times 100$$

The ninth statistic tool is the cost function (CF), which indicates the correlation, the best line of fit between the simulated and actual measurements.

(10) $$\mathrm{CF}=\frac{1}{N}\sum_{n=1}^{N}\frac{\left|D_{n}-M_{n}\right|}{\sigma D}$$

The tenth statistic tool used is the index of agreement (IOA), which estimates the fitness of model prediction. IOA is a dimensionless quantity and varies between 0 and 1. As the value of IOA approaches closer to 1, the prediction accuracy increases.

(11) $$\mathrm{IOA}=1-\frac{\sum_{n=1}^{N}{\left(D_{n}-M_{n}\right)}^{2}}{\sum_{n=1}^{N}{\left(\left|D_{n}-{\bar{D}}_{n}\right|+\left|M_{n}-{\bar{M}}_{n}\right|\right)}^{2}}$$

Here, *D_n_* is the measured data, *M_n_* is the simulated data, *N* is the sum of all the matching data between the model and measurements, n is the nth comparison, and *σD* is the standard deviation of the measured data.

### 2.6. Water Quality Index (WQI) Development

WQI is a comparative number which mirrors the combined impacts of various water quality variables on the general quality of the specific water sampled. The indices simplify different qualifications based on the individual parameter value into a single composite value which is easier to use and understand. Globally, the World Health Organization (WHO) has developed WQI for different uses such as the WQI for drinking. Various governmental and regional authorities have developed their own WQI based on their priorities, levels of technology, and institutional capacities. There are some other WQI methods, but weighted arithmetic index also allows use of secondary data without conducting any field experiment and household investigations from different correspondents. The benefits of the weighted arithmetic mean method applied in the study are as follows [91,92,93].

- This technique integrates information from several water quality variables into a numerical form that measures the fitness of the water ecosystem with the number scale.
- Fewer variables are needed in evaluating the overall water quality for specific use.
- Advantageous for the report of overall water bodies health for the corresponding community and policymakers.
- Mirrors the overall influence of different water quality variables that are significant for the management and administration of water environments.

The main method for deriving WQI is the weighted arithmetic index (WAI) method [46,92,93]. The WQI was derived from the WAI method, proposed by Horton [94] and formulated by Brown [95]. The weighted arithmetic based WQI is derived from the following expression.

(12) $$\mathrm{WQI}=\sum_{i=1}^{k}W_{i}\times Q_{i}$$

(13) $$\sum_{i=1}^{k}W_{i}=1$$

Here, *k* is the count of parameters, *W_i_* is the value reflecting the unit weight of each water quality parameter, and *Q_i_* is the water quality rating of each variable. The unit weight of water quality variable is inversely proportional to the standard limits of the corresponding water quality variable. *Q_i_* was determined according to the formula below established by Brown [95].

(14) $$Q_{i}=100\left[\left(O_{i}-I_{i}\right)/\left(P_{i}-I_{i}\right)\right]$$

where *O_i_* indicates an observed value of each parameter, *P_i_* is the standard allowable value of each variable, and Ii is the ideal value of each parameter in a clean ecosystem. With the exception of pH and dissolved oxygen, all the ideal values (*I_i_*) are assumed to be zero for drinking water [96]. The sub-index of each water quality parameter is obtained by multiplying the *Q_i_* rating with its unit weight. Using another WQI Calculator available online, the results were confirmed for different geo-graphical locations (https://www.water-research.net). Table 1 describe the ranges and status of WQI.

The study has used basic physiochemical parameters such as BOD, DO, pH, and NO3 as main indicators for WQI assessment. The adopted parameters are mostly used as water quality indicators in many countries and are therefore more consistently monitored. Therefore, they were selected as performance indicators to examine the water quality profile. Furthermore, WQI index can be developed with many water quality parameters based on their availability. In this study, we are dealing only with those parameters which are common in all streams. Since, the online WQI calculator does not have the total nitrogen or inorganic nitrogen, in order to compare our results with the online available calculator, we utilized the relevant available and common parameters.

## 3. Results and Discussion

### 3.1. Model Calibration and Validation

**Figure 4:**
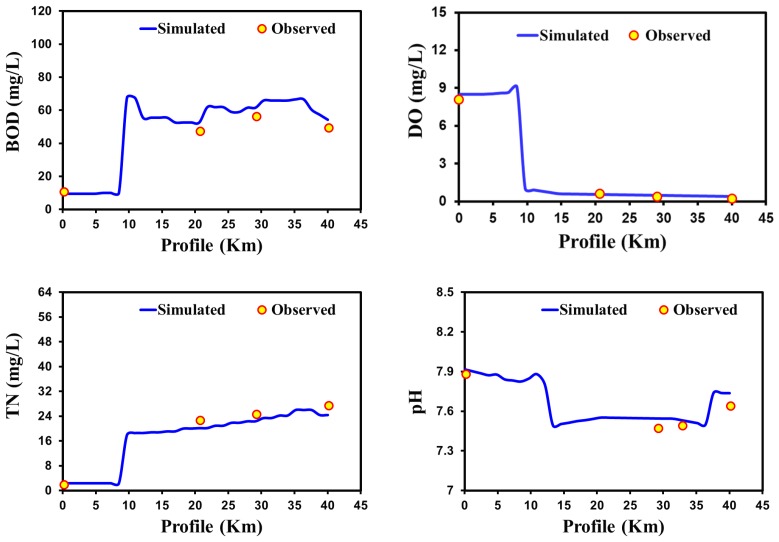
Confirmation of water quality model results in Yamuna River (arid climatic region) for BOD, DO, TN, and pH.

**Figure 5:**
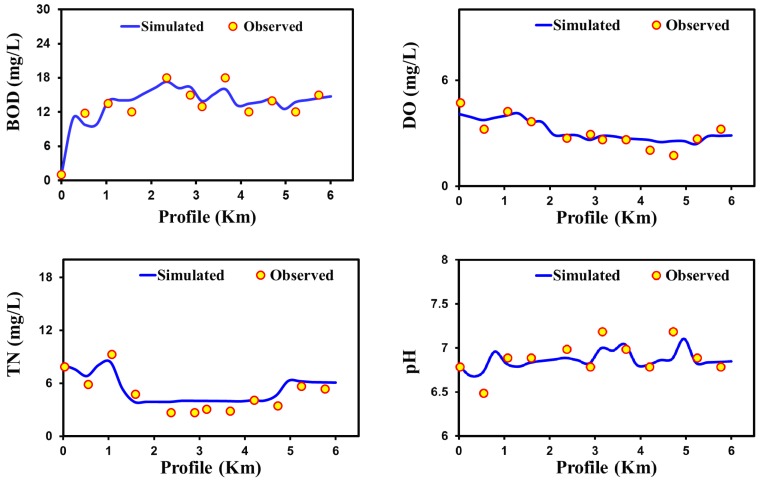
Confirmation of water quality model results in Galing River (tropical climatic region) for BOD, DO, TN, and pH.

**Figure 6:**
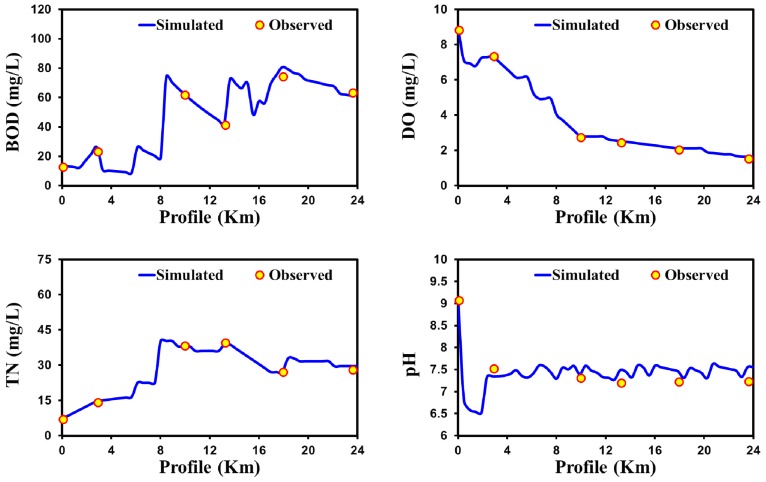
Confirmation of water quality model results in Baghmati River (temperate climatic region) for BOD, DO, TN, and pH.

**Figure 7:**
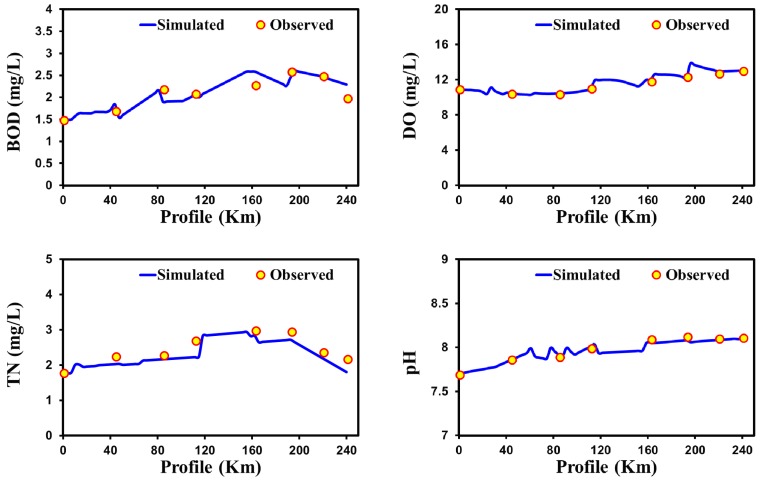
Confirmation of water quality model results in the Nakdong River (cold climatic region) for BOD, DO, TN, and pH.

The performance of the QUAL2Kw model has been assessed based on the simulated and observed results of BOD, DO pH, and nitrogenous compound at their corresponding monitoring stations on each river over different climatic regions of Asia. Summaries of the calibrated and confirmed water quality profiles and statistical evaluations are shown in Figure 4, Figure 5, Figure 6 and Figure 7 and Table 2. The simulated value of the model’s output showed good agreement with the observed value via different statistical approaches to assess the performance of the modeling tools for different climatic regions. To attain a solid statistical evaluation of model performance, 10 different statistical analyses were performed. Figure 4 shows the output profile of the validated values for BOD, DO, TN, and pH for the case of Yamuna River. Once the Yamuna River enters the urban region, the water quality degrades in terms of BOD, TN, and DO. The result indicates that the Yamuna waterway health is very poor according to local water quality standards. The spatial profile of the Yamuna River water quality concurs with the findings of previous studies [97,98,99]. Moreover, study included all of the possible inflow sources contributing to the mainstream river with their input concentrations of BOD, DO, pH, and other nitrogenous compounds. Previous studies have only defined the water quality based on BOD and DO by not considering pH and total nitrogen, which also have directly impact on the overall water quality [74,97,98].

The statistical analysis in the Table 2 shows that the QUAL2Kw performed well to some extent for the Yamuna River in arid climatic regions. All of the simulated water quality parameters (BOD, DO, TN, and pH) show the lower error value of MAE, MSE, RMSE, NRMSE, and MAPE for the observed values using the QUAL2Kw model in the Yamuna River (Table 2). In the validation, all the statistical errors (MAE, MSE, RMSE, NRMSE, and MAPE) have lower values (between simulated and observed) for the simulated water quality parameters such as DO, BOD, TN, and pH (Table 2). Similarly, predictions of model have higher the Pearson correlation coefficient value with observations. Furthermore, statistical analysis employing ME, PMB, CF, and IOA revealed that the model shows better appraisal (Table 2). These statistical results comparing simulated and observed values showed that the model is nearly consistent in predicting the river water quality profiles. Overall, the simulated results reveal that the quality of Yamuna river is very bad comparative to World Health Organization (WHO) standards. Our results are parallel with the previous study [92], which applied QUAL2Kw for assessment of the Yamuna River water quality. Moreover, this study reveals that significant changes were observed in DO profile in the arid climatic region. An arid climate usually has a warm temperature and the DO level reduced rapidly due to more consumption by river biology and less reoxygenation [100].

Figure 5 shows the result of validated profiles for BOD, DO, TN, and pH for the case of Galing River. The study assessed that the health of the waterway is very poor in accordance with the National Water Quality Standards (NWQS) for Malaysia. For example, the spatial profile for BOD and DO varied from 10 to 22 mg/L Class II–III and 1.85–4.5 mg/L (Class V–III), respectively. The findings of previous studies also revealed that the quality of the waterway is very poor [78,79]. All of the simulated variables (BOD, DO, TN, and pH) show the lower error value of MAE, MSE, RMSE, NRMSE, and MAPE with observing values for the QUAL2Kw model. In the validation (Figure 5), statistical estimators (MAE, MSE, RMSE, NRMSE, and MAPE) has also shown lower values (among predicted and observed) for BOD, DO, TN, and pH (Table 2).

Figure 6 shows the results of validated profiles for BOD, DO, TN, and pH of Bagmati River. The validated results (Figure 6) show that the water quality profile in the initial 10 km, differ from the rest of the downstream profile. The downstream water is extremely polluted and does not even meet the marginal standard of water quality. In the upstream segments of the river, the DO level is still near to 6 mg/L, indicating comparatively better quality. As the water flows toward downstream, it becomes more polluted due to mixing with household waste discharge and cremation activities. Moreover, decayed flowers contribute to the pollution discharge in the stream from offerings made by worshippers at Pashupatinath Temple.

Furthermore, the cremation activities performed along the bank of the river contribute to the pollution discharge. The high value of BOD and TN concentrations for a stretch of the river from 10 to 24 km was due to the inflow from the highly contaminated Dhobi, Tukucha, Hanumante, and Bishnumati kholas drains. The validated results (Figure 6) show that the QUAL2Kw model shows good agreement with the observed data. Statistical errors such as MAE, MSE, RMSE, NRMSE, and MAPE show the lower values between the predicted and observed values for pH, DO, BOD, and TN (Table 2).

Figure 7 shows the validated longitudinal profile of Nakdong River water quality for BOD, DO, TN, and pH. Minor changes are apparent in the spatial variation of water quality for all water quality variables, despite the very long profile selected in this study of around 250 km. The water quality profile for DO shows that the cold climate has a comparatively higher reaeration of oxygen level than any other climatic regions. The smaller variation and rising of the DO profile toward downstream might be due to the positive effect of temperature and pressure on the actual amount of oxygen in the cold climatic region [100]. Furthermore, passing near urbanized regions increased the amounts of BOD and TN in the river but did not decline the amount of DO. Statistical error estimators such as MAE, MSE, RMSE, NRMSE, and MAPE show a lower value for the QUAL2Kw model between the predicted and observed values for pH, DO, BOD, COD, and TN (Table 2).

### 3.2. Models Accuracy Assessment

In this study, the water quality data of two different events were used. The first event data were used to calibrate the models by comparing the model prediction and mainstream observation values with statistical evaluation (Table 2) to obtain some reasonable results. The environmental parameters including kinetics and stoichiometric constants were adjusted to obtain the reasonable profile of river water quality (Tables S1–S4). Table 2 shows the goodness of fit of the calibrated models for MAE, MSE, RMSE, NRMSE, MAPE, PCC, ME, PM Bias, CF, and IOA. Therefore, the calibrated model’s parameters, including kinetics and stoichiometric constants, were applied to validate the model.

### 3.3. Assessment of Water Quality Index Using Validated Results

**Figure 8:**
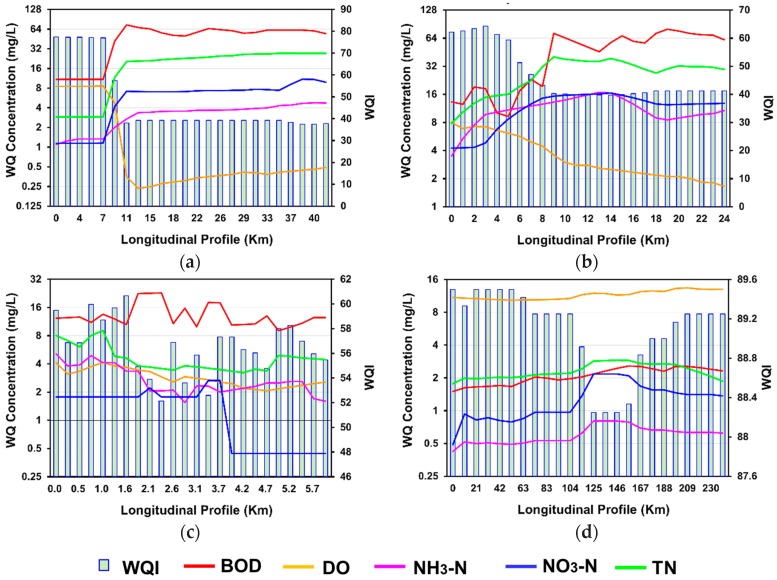
Spatial effect of water quality concentration on the overall water quality index (**a**) Yamuna River, (**b**) Baghmati River, (**c**) Galing River, and (**d**) Nakdong River.

**Figure 9:**
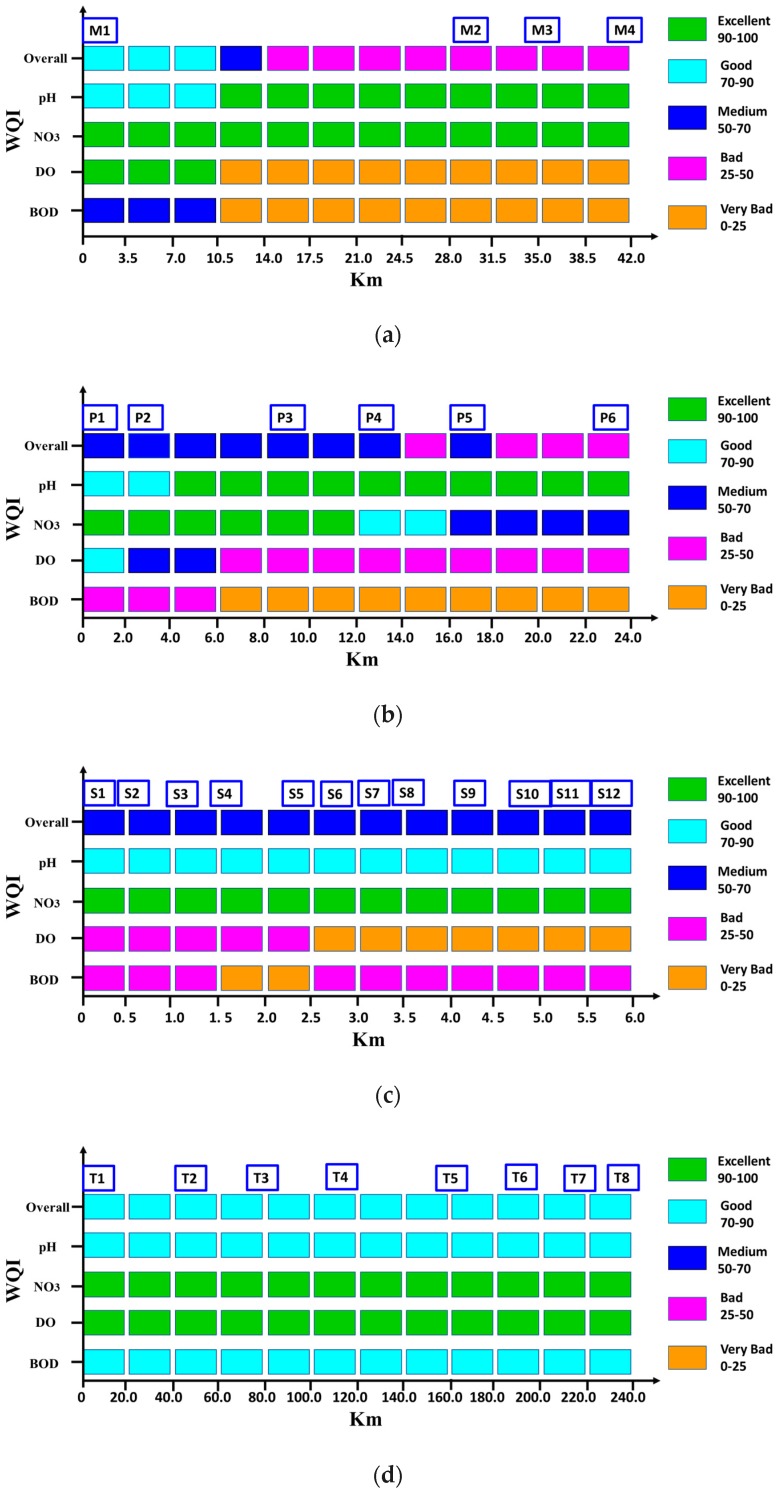
Spatial profile of water quality index: (**a**) Yamuna River India/arid climate, (**b**) Baghmati River Nepal/temperate climate, (**c**) Galing River Malaysia/tropical climate, and (**d**) Nakdong River Korea/cold climate.

For comparison purposes, in this study each length of the spatial profile was divided into an equal number of WQI segments. Figure 8 presents the spatial effects of the water quality concentration on the spatial profile of WQI. Figure 8 shows that, overall, the concentrations of BOD, NH_3_, NO_3_, and TN have directly influenced on the spatial profile of WQI, with the exception of DO concentration, which was increased. The correct level of DO concentration is desirable for a sustainable water ecosystem. The overall water quality spatial profile of the Yamuna River ranged from ‘good’ to ‘poor’, and the mean value was close to the poor water quality (Figure 9a). The WQI profile of Bagmati River ranged from ‘medium’ to ‘poor’, and the mean value was close to the medium water quality (Figure 9b). The WQI profile of Galing River was ‘medium’, and less spatial variation was observed for the overall WQI profile (Figure 9c). However, spatial variation was still observed in the fitness levels of the WQI for the case of the BOD and DO sub-indexes.

These results indicated that the water quality of the Yamuna, Baghmati, and Galing Rivers is severely affected by the waste water direct discharge and climatic warming conditions [101]. Furthermore, along with the point and non-point source urban discharge, the concentrations of TN, DO, and BOD were also affected by the environmental and climatic conditions. The DO level shows a more substantial decline in the warm climatic region such as the arid, tropical, and temperate climates. As the water merges with the residential discharge, the DO is rapidly consumed in the relatively warmer climatic zones due to elevated oxidation process during the degradation of the organic matter [100]. This concurs with a previous study by Blumberg and Toro [101], which shows the climatic influences on the concentration of the DO profile along with other house pollution discharge.

### 3.4. Spatial Scale Interrelationship between Water Quality Parameters and Flow Profile

**Figure 10:**
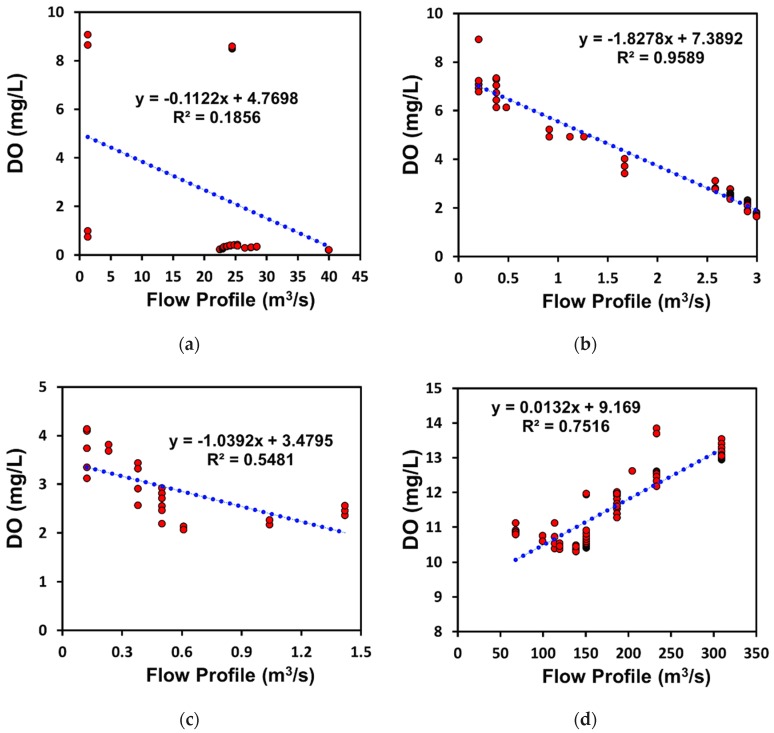
Dissolved oxygen concentration with respect to flow profile (**a**) Yamuna River, (**b**) Baghmati River, (**c**) Galing River, and (**d**) Nakdong River.

Figure 10 shows the spatial scale interrelationship among the quantity of the river flow profile and the concentrations of BOD and DO. Usually, a high fresh-water flow rate is considered to improve the concentration of DO. However, exceptions occur since the concentration of DO, BOD, and nitrogen also depends on the different reaction rates, which differ among the climatic zones. This study shows that the concentration of DO increases in the cold climatic zone with respect to the flow rate, while DO concentration declines in all other climate classes including the arid, temperate, and tropical zones with respect to the flow profile. The greater possibility of a high reoxygenation rate is also observed in the cold climatic zone than in the other climate regions (Figure 10d). Although the longest possible stretch of the Nakdong River was selected, the DO reoxygenation seems to be greater in the cold climatic region than in all other climatic zones. The profile of river flow stated to as quality versus quantity in the magnitude of longitudinal regime assessment. The concentration of river pollution comprises different nutrients in the water ecosystem [102]. Overall, the BOD profile trend is increasing in all climate classes which is due to addition of urban drain water in the river ecosystem (Figure 4, Figure 5, Figure 6 and Figure 7). Similarly, DO rate is declining in all climatic classes except cold climatic region. From this finding, it can be agreed that water quality is a function of both water natural climate as well as anthropogenic activities [13,14,15].

Understanding of the DO and stream temperature is very important in aquatic biochemistry for sustainable riverine ecosystem [103,104,105]. Solubility of DO is inversely proportional to the temperature, therefore higher stream temperature reduces the oxygen solubility thereby depressing the DO. This is consistent with the observation that the instream DO in the warmer climates zone were lower compared to those in the cold climate zones. Best management practice for sustainable river water quality requires definition of the minimum DO for the survival of the moderate aquatic life and river ecosystem [106,107,108,109]. It is therefore important that different climate conditions, based on temperature variation, has to recognize the reduced solubility of DO in warmer climates as compared to the rivers in cold climatic zones. Hence a higher minimum DO level is required in the streams in the warmer climate than in those in the cold climate [106,107,108,109,110].

The solubility of DO operates on the principle of temperature effect on the kinetic energy of particles in a medium. Increasing temperature results in an increase of kinetic energy of the DO, breaking its intermolecular bonds, hence creating a tendency to escape from the liquid solvent. i.e., water. This study has confirmed this significance of this principle that rivers in warmer climate regions i.e., high surface and air temperatures, had lower DO and reaeration rates as compared to the river in the colder climatic zones. Since all the rivers were receiving waste effluents from urban discharges, the organic waste load carrying capacity of the rivers, for same flow depends on the resulting DO and reaeration rate. It is important for the municipal and water authorities to recognize the decreasing solubility of DO and increasing oxidation rate at higher temperature as a risk for the river in warmer climate zones [106,107,108,110,111,112].

Many features of water quality and quantity are systemically related. The health of a water ecosystem can spatially vary in importance depending on the actual quantity river flow profile, dilution rate, and chemical reaction constant rates in particular climatic zones. Environmental mechanisms are incorporated in the connection between the river flow and the health of subsequent water bodies. The objective of approaches such as water quality conservation (quantity and quality required to maintain water bodies at a safe level) is to safeguard the sustainability of the water environment [102]. Currently, most studies are concerned with the quantity of the flow to preserve the ecological health of a river [55], but do not focus on the effect of permanent climatic conditions which also has an influence on the sustainability of the water environment.

### 3.5. Study Significance and Limitations

This study is the first-time approach to describe the variation of surface water quality for the different climate classes of Asia. Previous studies by [4,5,6,7,8,9,10,11] described the effect of climate on individual stream water quality. However, no study has yet been conducted that describes the variation of water quality in different climate zones at a continental or global scale. In the current study, four different streams with similar characteristics in the major climatic zones of Asia were selected. Although the effect of anthropogenic activities on poor water quality is virtually unvarying, certain climatological conditions of different climate zones also deteriorate the water quality to below the required level for different purposes. Environmental events such as heavy rainfall and storms in the different climate classes lead to the extreme destruction of the embankments of streams, which in turn increases the concentration of nutrients in the water ecosystem. Environmental conditions can alter the rate of oxygenation, while deoxygenation leads to a variation in the streams’ water quality such as in DO, BOD, and nitrogenous constituents. According to the report by United Nations Environment Program [113], permanent environmental conditions in different zones may have a detrimental effect on river water quality, rendering the water unfit for drinking, irrigation, and other purposes. The major limitation of this study is the unavailability of temporally matched input data for model development. However, in the current study, the maximum possible matching of input data, at least for similar extents of year, was attempted. In addition, the current study only describes the spatial variation in the water quality profile. Furthermore, each climate zone has many streams. However, the study only considered rivers with the maximum possible environmental characteristics, enabling each stream to represent the main stream in each climate zone.

## 4. Conclusions

In the current study, the QUAL2Kw model is used to assess the water quality profile of selected streams in different climate zones of Asia. Different statistical analyses such as mean MAE, MSE, RMSE, NRMSE, MAPE, PCC, ME, PMB, CF, and IOA were applied to assess the performance of the model’s predictions. Furthermore, using validated results of each stream, spatial profile of the WQI index was developed. The overall WQI of Yamuna River was poor water quality. While Galing and Baghmati Rivers show medium water quality considering the WQI, the longest possible profile of the Nakdong River in the cold climate region showed no spatial variation in the overall status of the water quality index. The spatial profile of the WQI will lead to assist the decision-making process for the management of the water quality of water ecosystems. While Galing and Baghmati Rivers show medium water quality considering the WQI, the longest possible profile of the Nakdong River in the cold climate region showed no spatial variation of water quality.

All climatic streams, except Nakdong River (cold climatic stream), show a decline in DO as the water moves toward downstream areas. The cold climatic stream of Nakdong River shows an increase in DO, demonstrating high reoxygenation rates. The reach wise development of WQI using the best fit model can facilitate decision-making and could easily be implemented in other streams and lakes with similar characteristics in different climatological regions. Thus, surface water quality models such as QUAL2Kw used to assist WQI assessment offer a useful tool to efficiently predict the influences of contamination on stream water quality. The profile-based WQI assessment will be a valuable tool in an emerging management policy for improving water quality, rendering it easier for decision-makers to appraise different water quality management tactics. Furthermore, this research can be improved by conducting this study on other streams having similar characteristics and compare the results of this research. Moreover, the assessment of more than one river in each climate class having similar hydraulic and environmental characteristics will improve the overall findings of this study.

By comparing numerical results with analytical results will improve the overall soundness of this study. Using more than one stream from each climate class for assessment of regional climatic impact will provide a new direction in the sustainable management and assessment of regional ecology. Furthermore, in most of the cases, only anthropogenic activities on water quality are considered but the regional climatic impact has been ignored. The anthropogenic activities coupled with regional climatic impact will broaden the interaction of water quality variation with changing the environment, globally. The results of the present study can be useful in the monitoring and control of river water quality in different climate patterns. The outcomes obtained in this study will facilitate the development of a strategy for the viable improvement of sustainable water environments.

## Figures and Tables

**Table 1:** Ranges and status of WQI.

WQI	Water Quality Status (WQS)
90–100	Excellent
70–90	Good
50–70	Average
25–50	Poor
0–25	Very Poor

**Table 2:** Statistical evaluation of calibrated and validated results for predicted and observed.

River	Parameter	MAE	MSE	RMSE	NRMSE	MAPE	R^2^	ME	PMB	CF	IOA
Yamuna River Calibration	DO	0.45	0.25	0.50	0.21	0.67	0.90	0.86	−19.36	0.11	0.95
	BOD	12.28	172.1	13.12	0.31	0.23	0.86	0.84	−25.76	0.03	0.91
	TN	3.97	22.91	4.79	0.20	0.14	0.87	0.86	17.57	0.05	0.92
	PH	0.10	0.02	0.14	0.01	0.01	0.79	0.81	−0.83	0.21	0.92
Yamuna River Validation	DO	0.48	0.27	0.52	0.222	0.715	0.96	0.92	−10.6	0.12	0.97
	BOD	13.2	185	13.6	0.335	0.251	0.93	0.90	−17.7	0.03	0.93
	TN	4.32	24.9	4.99	0.221	0.149	0.95	0.93	19.1	0.05	0.94
	PH	0.11	0.02	0.14	0.016	0.014	0.87	0.89	−0.91	0.23	0.94
Baghmati River Calibration	DO	0.03	0.01	0.1	0.01	1.02	0.93	0.86	−0.29	0.01	0.96
	BOD	1.40	6.14	2.48	0.05	2.46	0.90	0.92	−0.80	0.49	0.92
	TN	0.73	1.04	1.02	0.03	2.10	0.90	0.89	−0.97	0.25	0.93
	PH	0.05	0.01	0.10	0.01	0.61	0.87	0.79	−0.60	0.02	0.93
Baghmati River Validation	DO	0.03	0.01	0.10	0.009	1.071	0.98	0.91	−0.30	0.01	0.97
	BOD	1.49	6.53	2.56	0.057	2.612	0.96	0.98	−0.85	0.52	0.93
	TN	0.78	1.12	1.06	0.035	2.255	0.97	0.96	−1.04	0.27	0.94
	PH	0.05	0.01	0.10	0.011	0.661	0.95	0.86	−0.65	0.02	0.94
Galing River Calibration	DO	0.25	0.12	0.35	0.12	0.11	0.87	0.73	−5.89	0.32	0.90
	BOD	1.46	3.52	1.88	0.11	0.09	0.90	0.90	8.66	1.84	0.91
	TN	0.44	0.27	0.52	0.20	0.19	0.84	0.43	17.57	0.56	0.88
	PH	0.05	0.02	0.14	0.01	0.01	0.72	0.79	−0.31	0.05	0.91
Galing River Validation	DO	0.27	0.13	0.36	0.124	0.115	0.93	0.78	−6.27	0.34	0.93
	BOD	1.57	3.78	1.94	0.122	0.094	0.97	0.97	9.31	1.98	0.94
	TN	0.48	0.29	0.54	0.216	0.202	0.91	0.47	19.1	0.61	0.91
	PH	0.05	0.02	0.14	0.009	0.007	0.79	0.87	−0.34	0.06	0.94
Nakdong River Calibration	DO	0.21	0.13	0.26	0.06	0.02	0.86	0.88	−0.93	0.05	0.96
	BOD	0.14	0.03	0.17	0.03	0.07	0.80	0.86	−1.02	0.07	0.92
	TN	0.22	0.07	0.26	0.10	0.09	0.86	0.86	9.09	0.13	0.93
	PH	0.11	0.02	0.14	0.02	0.01	0.65	0.84	0.18	0.06	0.93
Nakdong River Validation	DO	0.22	0.14	0.37	0.065	0.019	0.91	0.93	−0.98	0.05	0.97
	BOD	0.15	0.03	0.17	0.033	0.072	0.85	0.91	−0.49	0.07	0.93
	TN	0.24	0.07	0.26	0.112	0.096	0.93	0.92	9.77	0.14	0.94
	PH	0.12	0.02	0.14	0.017	0.015	0.71	0.91	0.20	0.06	0.94

## Supplementary Materials

The following are available online at http://www.mdpi.com/1660-4601/15/10/2258/s1, Table S1: Calibrated parameters for the QUAL2Kw model over Yamuna River in the arid climate region, Table S2: Calibrated parameters for the QUAL2Kw model over Baghmati River in the temperate climate region, Table S3: Calibrated parameters for the QUAL2Kw model over Galing River in the tropical climate region, Table S4: Calibrated parameters for the QUAL2Kw model over Nakdong River in the cold climate region.
