# Supplementary material for: Assessment of Water Quality Profile Using Numerical Modeling Approach in Major Climate Classes of Asia
Source: Int J Environ Res Public Health. 2018 Oct 15;15(10):2258. doi: 10.3390/ijerph15102258 (PMC6209875; doi:10.3390/ijerph15102258)
Supplement: Supplementary file 1 [file ijerph-15-02258-s001.pdf]

Table S1. Calibrated parameters for the QUAL2Kw model over Yamuna river in the arid climate region.

Parameter	Values	Units	Auto-Calibration	Min. Value	Max. Value
Carbon	40	gC	No	30	50
Nitrogen	7.2	gN	No	3	9
Dry weight	100	gD	No	100	100
ISS settling velocity	0.01	m/day	Yes	0	2
O2 reaeration model	Owens-Gibbs				
Slow CBOD hydrolysis rate	0.1	1/day	Yes	0.04	4.2
Slow CBOD oxidation rate	3.6	1/day	Yes	0.04	4.2
Fast CBOD oxidation rate	3.8	1/day	Yes	0.02	4.2
Organic N hydrolysis	0.10	1/day	Yes	0.02	0.4
Organic N settling velocity	0.06	m/day	Yes	0.001	0.1
Ammonium nitrification	5.2	1/day	Yes	0	10
Nitrate denitrification	1.53	1/day	Yes	0	2
Sed. denitrification transfer coeff.	0.56	m/day	Yes	0	1
Detritus dissolution rate	0.39	1/day	Yes	0	5
Detritus settling velocity	4.80	m/day	Yes	0	5
COD decay rate	0.58	1/day	Yes	0	0.8
COD settling velocity	0.79	m/day	Yes	0	1
First-order model carrying capacity	1000	mgA/m^2	No	1000	1000
Respiration rate	0.07	1/day	Yes	0.05	0.5
Excretion rate	0.12	1/day	Yes	0	0.5
Death rate	0.16	1/day	Yes	0	0.5
External nitrogen half sat constant	34.07	µgN/L	Yes	10	300
Inorganic carbon half sat constant	1.06E-05	Moles/L	Yes	1.30E−06	1.30E−04
Light model	half saturation				
Light constant	67.92	Langleys/day	Yes	1	100
Ammonia preference	67.23	µgN/L	Yes	1	100
Subsistence quota for nitrogen	1.45	mgN/mgA	Yes	0.0072	7.2
Maximum uptake rate for nitrogen	226.1	mgN/mgA/day	Yes	1	500
Internal nitrogen half sat ratio	4.06	-	Yes	1.05	5

Table S2. Calibrated parameters for the QUAL2Kw model over Baghmati river in the temperate climate region.

Parameter	Values	Units	Auto-Calibration	Min. Value	Max. Value
Carbon	42	gC	No	30	50
Nitrogen	7.12	gN	No	3	9
Dry weight	100	gD	No	100	100
ISS settling velocity	0.011	m/day	Yes	0	2
O2 reaeration model	Owens-Gibbs				
Slow CBOD hydrolysis rate	0.12	1/day	Yes	0.04	4.2
Slow CBOD oxidation rate	3.4	1/day	Yes	0.04	4.2
Fast CBOD oxidation rate	3.5	1/day	Yes	0.02	4.2
Organic N hydrolysis	0.11	1/day	Yes	0.02	0.4
Organic N settling velocity	0.07	m/day	Yes	0.001	0.1
Ammonium nitrification	5.3	1/day	Yes	0	10
Nitrate denitrification	1.52	1/day	Yes	0	2
Sed. denitrification transfer coeff.	0.58	m/day	Yes	0	1
Detritus dissolution rate	0.41	1/day	Yes	0	5
Detritus settling velocity	4.69	m/day	Yes	0	5
COD decay rate	0.59	1/day	Yes	0	0.8
COD settling velocity	0.79	m/day	Yes	0	1
First-order model carrying capacity	1000	mgA/m^2	No	1000	1000
Respiration rate	0.08	1/day	Yes	0.05	0.5
Excretion rate	0.11	1/day	Yes	0	0.5
Death rate	0.14	1/day	Yes	0	0.5
External nitrogen half sat constant	34.06	μgN/L	Yes	10	300
Inorganic carbon half sat constant	1.06E-05	Moles/L	Yes	1.30E−06	1.30E−04
Light model	half saturation				
Light constant	67.79	Langleys/day	Yes	1	100
Ammonia preference	67.22	μgN/L	Yes	1	100
Subsistence quota for nitrogen	1.39	mgN/mgA	Yes	0.0072	7.2
Maximum uptake rate for nitrogen	228.1	mgN/mgA/day	Yes	1	500
Internal nitrogen half sat ratio	4.08	-	Yes	1.05	5

Table S3. Calibrated parameters for the QUAL2Kw model over Galing river in the tropical climate region.

Parameter	Values	Units	Auto-Calibration	Min. Value	Max. Value
Carbon	40	gC	No	30	50
Nitrogen	7.3	gN	No	3	9
Dry weight	100	gD	No	100	100
ISS settling velocity	0.03	m/day	Yes	0	2
O2 reaeration model	Owens-Gibbs				
Slow CBOD hydrolysis rate	0.2	1/day	Yes	0.04	4.2
Slow CBOD oxidation rate	3.8	1/day	Yes	0.04	4.2
Fast CBOD oxidation rate	3.9	1/day	Yes	0.02	4.2
Organic N hydrolysis	0.12	1/day	Yes	0.02	0.4
Organic N settling velocity	0.07	m/day	Yes	0.001	0.1
Ammonium nitrification	5.3	1/day	Yes	0	10
Nitrate denitrification	1.56	1/day	Yes	0	2
Sed. denitrification transfer coeff.	0.58	m/day	Yes	0	1
Detritus dissolution rate	0.41	1/day	Yes	0	5
Detritus settling velocity	4.83	m/day	Yes	0	5
COD decay rate	0.61	1/day	Yes	0	0.8
COD settling velocity	0.81	m/day	Yes	0	1
First-order model carrying capacity	1000	mgA/m^2	No	1000	1000
Respiration rate	0.08	1/day	Yes	0.05	0.5
Excretion rate	0.14	1/day	Yes	0	0.5
Death rate	0.18	1/day	Yes	0	0.5
External nitrogen half sat constant	35.12	µgN/L	Yes	10	300
Inorganic carbon half sat constant	1.06E-05	Moles/L	Yes	1.30E−06	1.30E−04
Light model	half saturation				
Light constant	67.94	Langleys/day	Yes	1	100
Ammonia preference	67.71	µgN/L	Yes	1	100
Subsistence quota for nitrogen	1.44	mgN/mgA	Yes	0.0072	7.2
Maximum uptake rate for nitrogen	227.31	mgN/mgA/day	Yes	1	500
Internal nitrogen half sat ratio	3.98	-	Yes	1.05	5

Table S4. Calibrated parameters for the QUAL2Kw model over Nakdong river in the cold climate region.

Parameter	Values	Units	Auto-Calibration	Min. Value	Max. Value
Carbon	39	gC	No	30	50
Nitrogen	7.3	gN	No	3	9
Dry weight	100	gD	No	100	100
ISS settling velocity	0.01	m/day	Yes	0	2
O2 reaeration model	Owens-Gibbs				
Slow CBOD hydrolysis rate	0.12	1/day	Yes	0.04	4.2
Slow CBOD oxidation rate	3.8	1/day	Yes	0.04	4.2
Fast CBOD oxidation rate	3.9	1/day	Yes	0.02	4.2
Organic N hydrolysis	0.13	1/day	Yes	0.02	0.4
Organic N settling velocity	0.07	m/day	Yes	0.001	0.1
Ammonium nitrification	5.6	1/day	Yes	0	10
Nitrate denitrification	1.56	1/day	Yes	0	2
Sed. denitrification transfer coeff.	0.58	m/day	Yes	0	1
Detritus dissolution rate	0.41	1/day	Yes	0	5
Detritus settling velocity	4.83	m/day	Yes	0	5
COD decay rate	0.61	1/day	Yes	0	0.8
COD settling velocity	0.78	m/day	Yes	0	1
First-order model carrying capacity	1000	mgA/m^2	No	1000	1000
Respiration rate	0.12	1/day	Yes	0.05	0.5
Excretion rate	0.11	1/day	Yes	0	0.5
Death rate	0.10	1/day	Yes	0	0.5
External nitrogen half sat constant	34.1	µgN/L	Yes	10	300
Inorganic carbon half sat constant	1.06E-05	Moles/L	Yes	1.30E−06	1.30E−04
Light model	half saturation				
Light constant	67.97	Langleys/day	Yes	1	100
Ammonia preference	67.26	µgN/L	Yes	1	100
Subsistence quota for nitrogen	1.43	mgN/mgA	Yes	0.0072	7.2
Maximum uptake rate for nitrogen	227.3	mgN/mgA/day	Yes	1	500
Internal nitrogen half sat ratio	4.09	-	Yes	1.05	5
